# A Novel Notch-Related Gene Signature for Prognosis and Immune Response Prediction in Ovarian Cancer

**DOI:** 10.3390/medicina59071277

**Published:** 2023-07-09

**Authors:** Yanan Pi, Fusheng Sun, Zhaocong Zhang, Xiaoli Liu, Ge Lou

**Affiliations:** 1 Department of Gynecology, Harbin Medical University Cancer Hospital, Harbin 150086, China; 2 Harbin Obstetrics and Gynecology Hospital, Harbin Medical University, Harbin 150086, China

**Keywords:** ovarian cancer, Notch, NTRS, survival analysis, immunotherapy

## Abstract

*Background and Objectives:* Notch is a fascinating signaling pathway. It is extensively involved in tumor growth, cancer stem cells, metastasis, and treatment resistance and plays important roles in metabolic regulation, tumor microenvironment, and tumor immunity. However, the role of Notch in ovarian cancer (OC) has yet to be fully understood. Therefore, this study systematically described the expression, mutation, and copy number variation of genes in the Notch signaling pathway in OC and evaluated the relationship between gene mutation and Overall Survival (OS) prognosis. *Materials and Methods:* Notch risk score (NTRS) was established by univariate Cox regression analysis combined with Lasso regression analysis, and the efficacy of NTRS in predicting prognosis and immunotherapy response in patients with OC was verified. We further assessed the correlations of NTRS with clinical features, immune infiltration level, immune checkpoint expression, and immune characteristics. Additionally, differential expression and functions of the fourteen signature genes were confirmed via vitro assays. *Results:* The results showed that Notch genes (NTGs) were markedly differentiated between tumor and normal tissues, which may help to explain the high heterogeneity in the biological characteristics and therapeutic outcomes of human OC. A Notch risk (NTR) prognostic model based on 11 key NTGs was successfully constructed. Tumors with high Notch risk scores (NTRS) were independently associated with shorter overall survival and poorer immunotherapy outcomes. We further assessed the correlations of NTRS with immune characteristics. The results showed that NTGs play a key role in regulating the tumor immune microenvironment. Additionally, we validated the baseline and induced expressions of 14 prognosis-related NTGs in our own OC samples. In vitro assays confirmed that the knockdown of NCOR2 and APH1B and overexpression of HEY2 and SKP2 could inhibit the proliferation, invasion, and migration of OC cells. *Conclusions:* These findings emphasize that Notch multilayer changes are associated with the prognosis of patients with OC and the characteristics of immune cell infiltration. Our predictive signature may predict the prognosis and immunotherapy response of OC patients in an independent manner. NCOR2, APH1B, HEY2, and SKP2 may more prominently represent important indicators to improve patient prognosis.

## 1. Introduction

Ovarian cancer (OC) is a common gynecological malignant tumor that ranks second in morbidity and first in mortality among gynecological malignant tumors. According to data from the 2020 Global Cancer Incidence, Mortality, and Prevalence, approximately 313,959 people were diagnosed with OC globally in 2020, accounting for 3.4% of all new cancer cases and 4.7% of all new cancer deaths [1]. The onset of OC is insidious. Nearly 80% of patients are diagnosed at advanced stages, and the 5-year survival rate is less than 40% [2]. The development of approved diagnostics and treatments for OC has not changed considerably compared with those for other cancers. The use of antiangiogenic therapy and polyadenosine diphosphate ribose polymerase inhibitors in maintenance therapy increases progression-free survival (PFS) in patients with epithelial OC but does not extend OS [3,4]. Immunotherapy has become the focus of current research as a promising and effective maintenance therapy. Although immune checkpoint inhibitors (ICIs) can produce a lasting response in some patients, OC as an immunogenic tumor is substantially heterogeneous and complex, and patients with OC show individual differences in response to treatment and prognosis [5]. Therefore, identifying effective prognostic biomarkers for OC is of great importance for improving the survival rate of patients and accelerating the development of precision therapy.

The tumor microenvironment (TME) has a wide range of implications for tumorigenesis because it hosts tumor cells, interacts with surrounding cells through the circulatory and lymphatic systems, and interacts with the immune system and modified extracellular matrix to influence cancer development and progression [6,7,8,9,10]. The Notch signaling pathway, as a relatively conserved cellular signal, is a key factor that determines cell fate in multiple organ development and disease. This pathway is one of the major signaling pathways that mediate the direct contact between cells and regulate the apoptosis, proliferation, and differentiation of cell bodies [11,12]. Notch signaling is closely related to the TME and regulates tumor angiogenesis and immune cell infiltration. This pathway contributes to the development of T and B lymphocytes to a large extent [13,14]. In head and neck squamous cell carcinoma (HNSCC), the high levels of Notch1 receptors are associated with the high infiltration of CD163+ tumor-associated macrophages [15]. The abnormal activity of the Notch signaling pathway is associated with the occurrence and development of various cancers and plays an important role in regulating stem and progenitor cells [16]. Notch also plays different carcinogenic and tumor-suppressive roles owing to the heterogeneity of the TME. The Notch pathway is an important therapeutic target for many cancer types. Wang et al. found that Notch signaling pathway mutations may be a promising biomarker for immune checkpoint-blocking therapy in patients with colorectal cancer (CRC) [17]. Mutations in the Notch signaling pathway are associated with increased tumor-specific CD8+ T cell count, decreased inhibitory regulatory T cell count, enhanced antitumor immunity, early disease occurrence, and lower metastasis rates in patients with CRC [17]. In non-small-cell lung cancer (NSCLC), patients with high mutations in Notch signaling have remarkably improved PFS and OS. High mutations in Notch signals may be used as a biomarker to predict the prognosis of patients with NSCLC treated with ICIs [18]. In conclusion, a comprehensive analysis of NTGs can help identify novel biomarkers, predict sensitivity to therapy, and develop new therapeutic targets. Further evidence initially confirmed that NOTCH prognostic features could be used to predict the prognosis of OC [19]. However, there is still a need to further identify more accurate prognostic-related genes to predict patient outcomes and increase the development of novel immunotherapies.

Herein, the expression, mutation, and copy number variations (CNVs) of NTGs in OC specimens were first identified from The Cancer Genome Atlas (TCGA) and Gene Expression Omnibus (GEO) databases by integrating NTGs. Then, the relationship between gene mutation and OC prognosis was evaluated. An NTR prognostic model was established, and the relationships of NTRS with prognostic, clinical, and immune-related characteristics were analyzed. Surprisingly, the high and low NTRS groups had unique immune cell infiltration characteristics. The results suggest that changes in the NTG multilayer are related to the prognosis of patients with OC and play an important role in immunotherapy.

## 2. Materials and Methods

### 2.1. OC Datasets and Preprocessing

Data were collected from five public cohorts (the expression matrix was transformed to transcripts per million). Sample expression data and clinical information were obtained from TCGA (https://xenabrowser.net/datapages/, accessed on 3 October 2021), Genotype-Tissue Expression (GTEx, https://xenabrowser.net/datapages/, accessed on 3 October 2021), GEO (https://www.ncbi.nlm.nih.gov/geo/, accessed on 11 October 2021), and International Cancer Genome Consortium (ICGC, https://dcc.icgc.org/projects/, accessed on 11 October 2021). Information about immunotherapy was obtained from IMvigor210CoreBiologies. Protein-coding genes were selected from the TCGA-GTEX data, and the intersection of genes in the GEO and ICGC data sets was obtained. The KEGG NOTCH SIGNALING PATHWAY, HALLMARK NOTCH SIGNALING, BIOCARTA NOTCH PATHWAY, PID NOTCH PATHWAY, and REACTOME SIGNALING BY NOTCH gene sets were downloaded from MSIGDB for summarizing. The intersection of pathway genes and public cohort database genes was analyzed.

### 2.2. Identification of Differentially Expressed NTGs and Assessment of Their Ability to Distinguish Biological Groups

Differentially expressed NTGs were screened in tumor and normal tissues using the limma package in R with |log2FC| > 1 and *p* < 0.05 as the thresholds. Volcanic and heat maps were constructed to display the differentially expressed NTGs. The NTGs were then evaluated for their ability to distinguish biological groups in ovarian tumor samples. Principal component analysis (PCA) was performed on all samples based on the expression values of all NTGs to further analyze the role of NTGs in the biological grouping of samples.

### 2.3. Delineating the Genome-Level Variations in NTGs and Their Effect on Prognosis

NTGs were displayed at the mutation level using the MAfTools package in R, and the mutation situations of these genes were described at the overall level. The relationship between NTG mutation and OS was further analyzed. Similarly, the CNVs of NTGs in the whole sample of OC were also displayed and grouped according to whether CNV occurred, and the differential expression of NTGs between groups was also displayed.

### 2.4. Screening and Analysis of Prognosis-Related NTGs

Based on univariate Cox regression analysis, the NTGs that were significantly correlated with OS prognosis of ovarian cancer were screened (*p* < 0.05) and displayed as a whole by forest map and individually as the Kaplan–Meier (KM) curves of individual genes.

### 2.5. Construction and Validation of a Notch-Related Prognostic Risk Model

Lasso regression analysis was performed based on the selected prognostic NTGs to prevent redundancy and construct prognostic-related NTR models. The calculation formula of NTRS is as follows:

NTRS = ∑ (*gene_i_* × *coef_i_*),

where *gene_i_* is the expression level of key genes after Lasso regression, and *coef_i_* is the weight.

The formula was also used to calculate the risk score in the validation set. The median value or Surv Cutpoint function was used to determine the optimal threshold points for the high and low NTRS groups in each dataset. KM survival analysis was used to evaluate the predictive power of the prognostic model.

### 2.6. Correlation between NTRS and Prognosis after Immunotherapy

The IMvigor210CoreBiologies dataset (348 samples, including 203 samples with bladder cancer data only and 168 samples with immunization data) was used to calculate NTRS. The samples were grouped using the median NTRS as the threshold to verify the utility of NTRS in the immunization dataset.

### 2.7. Correlation Analysis of NTRS with Clinical Features and Immunity

Whether significant differences in NTRS exist among different clinical characteristics was analyzed by the Wilcoxon test. CIBERSORT was then used to calculate the abundance of immune cells in tumor samples and analyze whether differences in immune cells exist between the high and low NTRS groups (Wilcoxon test). The tumor score, stroma score, and tumor purity of the tumor samples were predicted using ESTIMATE, and the levels of different immune characteristics were calculated using single-sample gene set enrichment analysis (ssGSEA). Spearman correlations between these characteristics and NTRS were calculated and presented. Finally, a box plot was drawn to show the differences in immune checkpoint gene expression between the high and low NTRS groups (Wilcoxon test).

### 2.8. Quantitative Real-Time PCR

We obtained OC tissue samples and normal precursor tissue samples (fallopian tube, FT) from 12 OC patients who underwent cytoreductive surgery at Harbin Medical University Cancer Hospital. Trizol (Sigma, St Louis, MO, USA) was used to extract total RNA. According to the previous protocols followed by cDNA synthesis with PrimeScript^TM^ RT Master Mix (RR036A, TAKARA, Kusatsu, Japan). mRNA expression levels were measured by TB Green^®^ Premix Ex Taq^TM^ II Kit (RR820A, TAKAR) on Applied Biosystems StepOnePlus. Melting curve analyses were performed, and Ct values were determined during the exponential amplification phase of real-time PCR. The 2^−ΔΔCt^ method determined relative fold changes between tumor and FT tissues.

### 2.9. Colony Formation and Cell Proliferation Assay

A2780 cells transfected with HEY2 and SKP2 and OVCAR3 cells transfected with NCOR2 and APH1B were seeded into the six-well plate with 100 cells per well and cultured for about 14 days. When the cell colony stopped culturing, paraformaldehyde was used to fix the cells for 30 min. After gentle washing, crystal violet was used to dye the cells. Excess crystal violet was removed with purified water for half an hour and quantified by ImageJ software after being photographed. Cell viability was measured by the Cell Counting Kit-8 (CCK8) assay. OC cells were plated into 96-well plates for 24 h, 48 h, 72 h, 96 h, and 120 h, followed by adding 100 µ CCK8 reagent (Biyuntian, Shanghai, China) in a 5% CO_2_ incubator at 37 °C for 2 h. Optical densities were measured using a microplate reader (Thermo Fisher Scientific, Somerville, MA, USA) at wavelengths of f 450 nm (OD450) to determine cell proliferation.

### 2.10. Invasion and Migration Assays

In the invasion test, the upper chamber was coated with Matrigel (Corning, NY, USA), and the lower chamber was added to 800 µL of medium containing 10% fetal bovine serum (FBS). The prepared cells were added to the upper chamber at the dosage of 5 × 10^4^ and cultured for 24–48 h. The cells in the upper chamber were removed. Finally, the lower surface cells were fixed with 4% paraformaldehyde, stained with crystal purple, photographed, and counted under the microscope. Cell migration was measured using a wound-healing assay. OVCAR3 and A2780 cells (1 × 106) were inoculated in six-well plates overnight. A 200 μL pipette tip was used to make a series of perpendicular scratches. Cells were then washed three times with PBS, and the basal medium was added. The scratch healing was recorded at 0 h and 48 h with a microscope.

### 2.11. Statistical Analyses

All statistical analyses were managed by R software (Version 3.6.3) and SPSS (Version 25). The unpaired *t*-test was used to compare the means of two groups, and a two-tailed *p*-value of <0.05 was considered statistically significant.

## 3. Results

### 3.1. Defining the Expression of NTGs and Their Role in Distinguishing Biological Groups in OC

**Figure 1:**
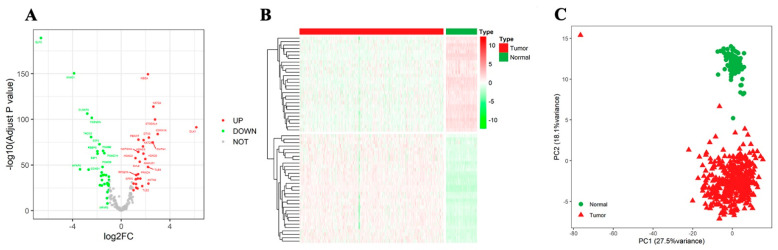
Identification of differentially expressed NTGs. (**A**) Volcano map of differential NTGs. (**B**) Heatmap of differential NTGs in OC. (**C**) PCA analysis.

Sixty differentially expressed NTGs were screened from OC tumor tissues and normal tissues. Among these, 28 genes were highly expressed in OC tissues, and 32 genes were highly expressed in normal tissues. PCA was performed on all samples in the database based on the expression values of the NTGs. The results showed that NTGs could indicate the biological grouping of samples (Figure 1).

### 3.2. Landscape of Genetic Variation of NTGs and Correlation with Prognosis in OC

**Figure 2:**
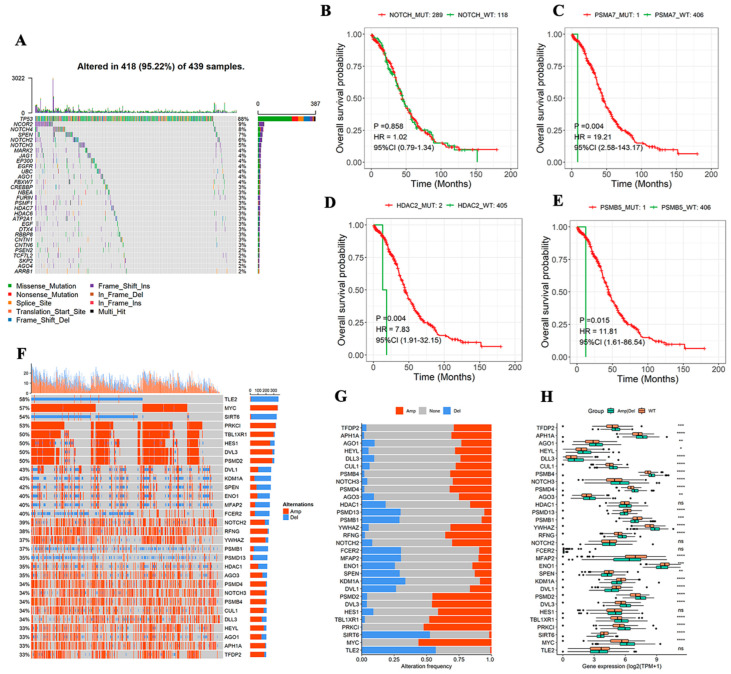
Characterization of NTGs in tumors at the biological level. (**A**) Genomic changes of 439 OC samples from TCGA, mutations of top 20 genes with mutation rate. (**B**) Relationship between global mutation and OS. (**C**–**E**) Relationship between single NTG mutation and OS. (**F**) CNVs of top 30 genes. (**G**,**H**) NTG expression between groups with or without CNV. * *p* < 0.05, ** *p* < 0.01, *** *p* < 0.001, **** *p* < 0.0001.

The MAfTools package in R was used to display the mutation level of the top 20 NTGs and depict the mutation situation of these genes from the overall level. TP53 had the highest mutation rate (88%), followed by NCOR2 and NOTCH4, with mutation rates of 9% and 8%, respectively (Figure 2A). The correlation between NTG mutation and OS prognosis was analyzed to further understand the relationship between NTGs and OC. The results showed no remarkable correlation between the overall mutation and OS prognosis (Figure 2B), whereas PSMA7, HDAC2, and PSMB5 mutations were remarkably correlated with OS prognosis. However, the mutation group sample was too small, and the results were meaningless (Figure 2C–E). Similarly, the CNVs of NTGs in the overall samples of OC (top 30) were shown. Most genes had some degree of amplification or deletion variation (Figure 2F). The final samples were grouped according to the presence of CNVs (Amp|Del versus WT). These genes had remarkable expression differences between groups (Figure 2G,H).

### 3.3. NTGs Predict OC Prognosis

**Figure 3:**
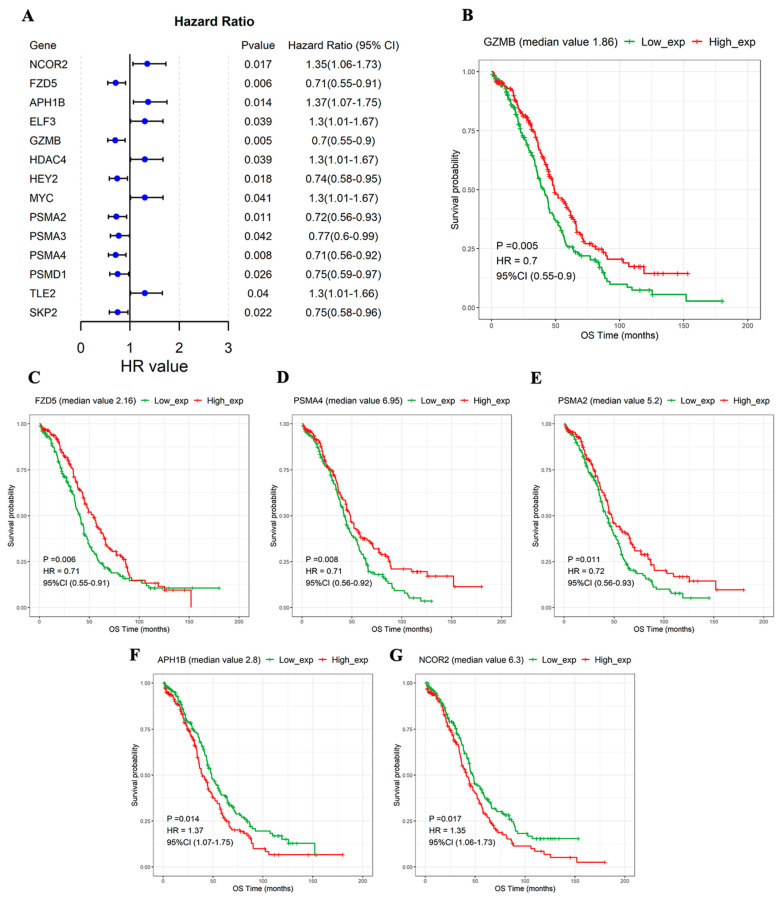
NTGs significantly associated with OS prognosis. (**A**) Forest map of 14 genes significantly associated with OS prognosis. KM curves in the high- and low-expression groups. (**B**) GZMB, (**C**) FZD5, (**D**) PSMA4, (**E**) PSMA2, (**F**) APH1B, (**G**) NCOR2.

The samples were divided into high- and low-expression groups according to the median expression value of the NTGs. Based on univariate Cox regression analysis, the NTGs remarkably correlated with OS prognosis in OC were screened (*p* < 0.05). The following 14 genes were significantly correlated with OS prognosis: nuclear receptor corepressor 2 (NCOR2), Frizzled 5 (FZD5), APH1B, ELF3, Granzyme B (GZMB), histone deacetylase 4 (HDAC4), HEY2, MYC, Proteasome subunit alpha type 2 (PSMA2), proteasome 20S subunit alpha 3 (PSMA3), Proteasome 20S subunit alpha 4 (PSMA4), Proteasome 26S subunit, non-ATPase 1 (PSMD1), the transducin-like enhancer of split 2 (TLE2), and SKP2 (Figure 3A). The KM survival curves of the 14 genes were drawn, and the NTGs with top 6 significance were displayed to more clearly describe the relationship between gene expression and prognosis. The group with high GZMB, FZD5, PSMA4, and PSMA2 expression had a remarkably better prognosis. Low expression APH1B and NCOR2 expression levels were associated with remarkably better prognosis (Figure 3B–G).

### 3.4. Risk Model Has Excellent Independent Prognostic Value

**Figure 4:**
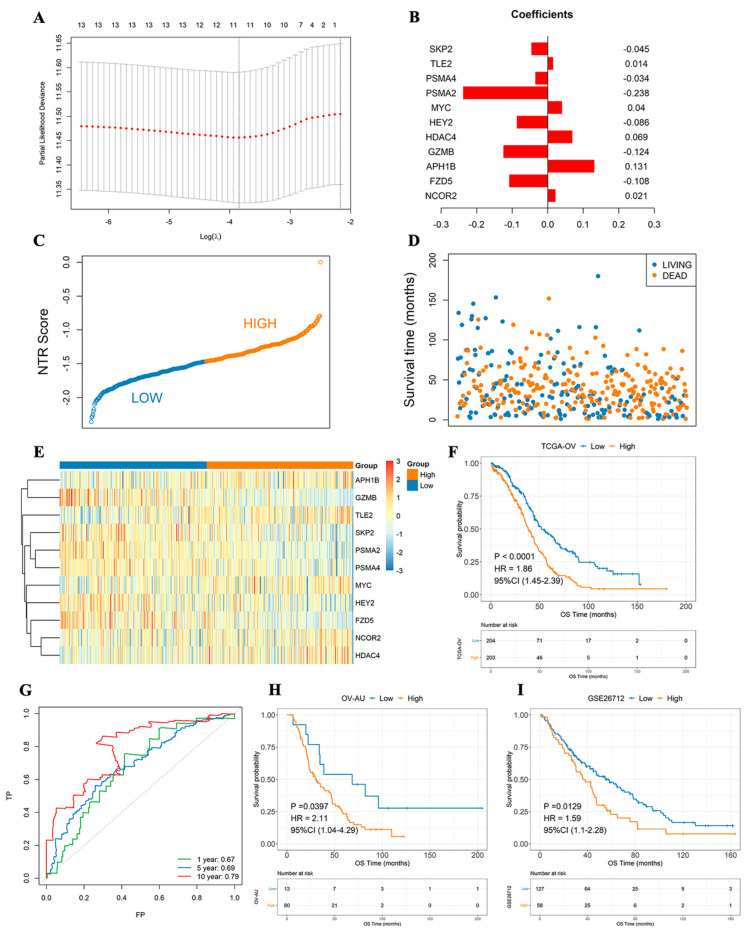
NTG-based prognostic signature constructed by Lasso–Cox regression analysis. (**A**) Lasso–Cox regression analysis. (**B**) Histogram of gene weight coefficient. (**C**) NTRS point figure. (**D**) Survival time of samples with different survival states. (**E**) Expression of model genes in high- and low-score groups. (**F**) Survival analyses of high- and low-score groups. (**G**) 1-, 5-, and 10-year ROC curves. (**H**) Survival analysis of OV–AU group with high and low NTRSs. (**I**) Survival analyses of medium and low NTRS groups from GSE26712.

The 14 prognosis-related NTGs obtained in the previous step were combined with Lasso regression analysis to further eliminate redundancy, and an NTR prognostic model composed of 11 NTGs was constructed (Figure 4A,B). According to the median NTRS, the samples were divided into high- and low-score groups. The results showed that the high-score group had a worse prognosis. The combined receiver operating characteristic (ROC) curve showed that the model was a good indicator for predicting the prognosis of patients, and its 10-year area under the ROC curve (AUC) was as high as 0.79 (Figure 4C–G). The formula was also used to calculate the NTRS of the two independent validation sets, and the surv_cutpoint function was used to determine the optimal threshold points of the samples in the high and low NTRS groups of the two data sets. The OS of the two groups was analyzed. Similarly, in both validation sets, the high-score group had a remarkably worse prognosis, whereas the low-score group had a substantially better prognosis (Figure 4H,I). The above analysis results show that the risk model has a good indicator effect on the clinical prognosis of samples and has good stability.

### 3.5. Age and NTRs Are Independent Prognostic Factors

**Figure 5:**
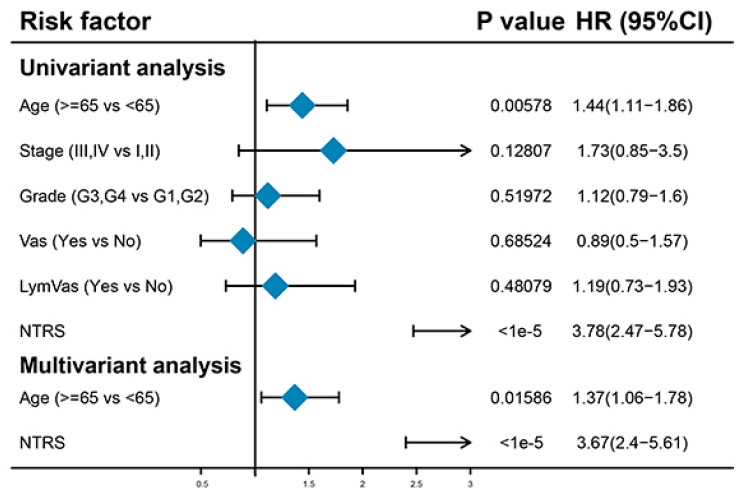
Univariate and multivariate regression analyses of clinical features and NTRs.

Univariate Cox regression analysis was performed to analyze the relationships of age, stage, grade, vascular invasion, lymphatic invasion, and NTRS with OS. Age and NTRS were remarkably correlated with OS. Multivariate Cox regression analysis based on these two features showed that age and NTRS were still substantially correlated with OS. This result indicates that age and NTRS can be used as independent prognostic factors (Figure 5). Next, age and NTRS were included in the construction of lipograms, and a new risk score was constructed. The indicative efficacy of the two models was evaluated by ROC curves, and the combination of the two models had the best prediction effect (Figure S1).

### 3.6. Role of NTRS in the Prediction of Immunotherapy Benefits

**Figure 6:**
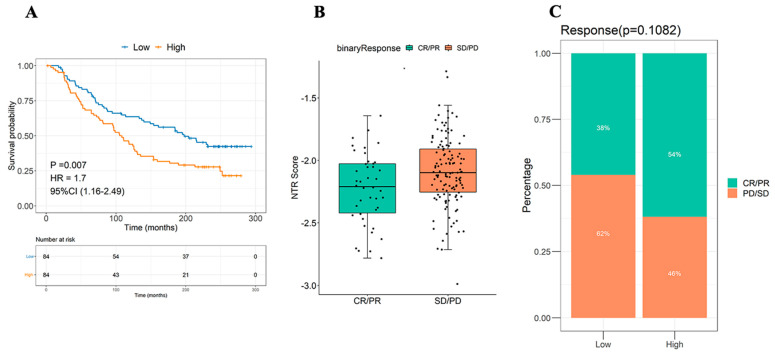
Efficacy of NTRS in the prediction of immunotherapy benefits. (**A**) Survival analyses of high- and low-NTRS groups. (**B**) Difference distribution of NTRS between CP/PR and SD/PD groups. (**C**) Composition of immune efficacy results in high and low NTRS groups.

NTRS was calculated using the IMvigor210CoreBiologies dataset, and 168 samples were divided into high and low NTRS groups according to the median NTRS to study the correlation of the high and low NTRS groups with the immunotherapy effect. Survival analysis revealed that high and low NTRS could be used to predict the prognosis of patients undergoing immunotherapy, in which a higher NTRS is associated with a poorer prognosis (Figure 6A). Subsequently, the NTRS of different therapeutic groups were compared. Remarkable differences in NTRS were found between the CR/PR and PD/SD groups (*p* = 0.011, Figure 6B,C).

**Figure 7:**
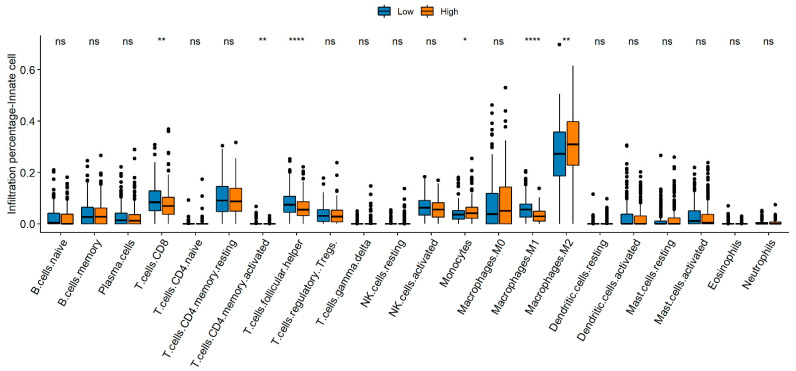
Comparison of immune cell composition between the high- and low-risk groups. * *p* < 0.05, ** *p* < 0.01, **** *p* < 0.0001.

**Figure 8:**
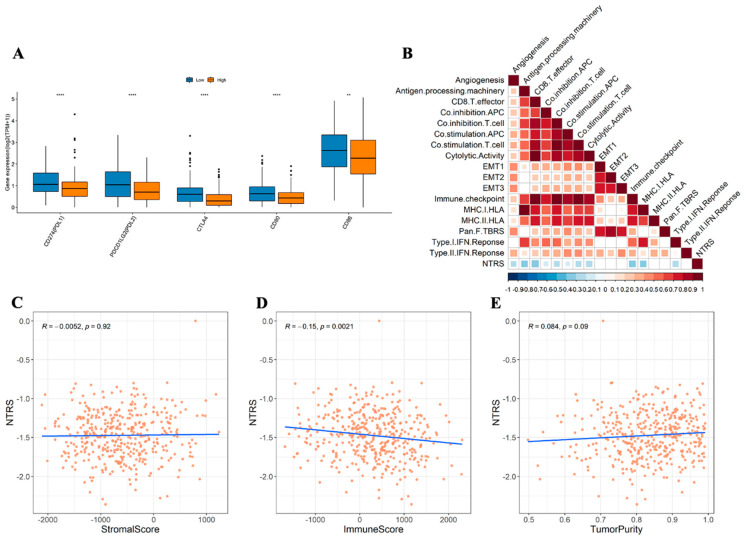
Correlation of risk score with immunity. (**A**) Distribution of immune checkpoint gene expression between high and low NTRS groups. (**B**) Correlation between NTRS and immune signature (blank area indicates insignificant correlation). Correlations of NTRS with. (**C**) stromal score. (**D**) immune score, and (**E**) tumor purity. ** *p* < 0.01, **** *p* < 0.0001.

CD8+ T cells, memory CD4 T cells, follicular helper T cells, and M1 macrophages were enriched in the low NTRS group, whereas monocytes and M2 macrophages mostly exist in the high NTRS group (Figure 7). The Wilcoxon test was used to analyze the differences in immune checkpoint genes between the high and low NTRS groups to further describe the correlation between NTRS and immunity. Five immune checkpoint-related genes were highly expressed in the low NTRS group (Figure 8A). Then, ssGSEA was used to calculate the levels of different immune characteristics, and the Spearman correlation and significance between these characteristics and NTRS were calculated. NTRS showed remarkable negative correlations with these characteristics (Figure 8B). Finally, ESTIMATE was used to predict the stroma score, immune score, and tumor purity of the tumor samples, and the Spearman correlation and significance between these features and the NTRS were calculated. NTRS was remarkably negatively correlated with the immune score (Figure 8C–E).

### 3.7. In Vitro Verification of Expression and Functions of 14 Key NTGs

**Figure 9:**
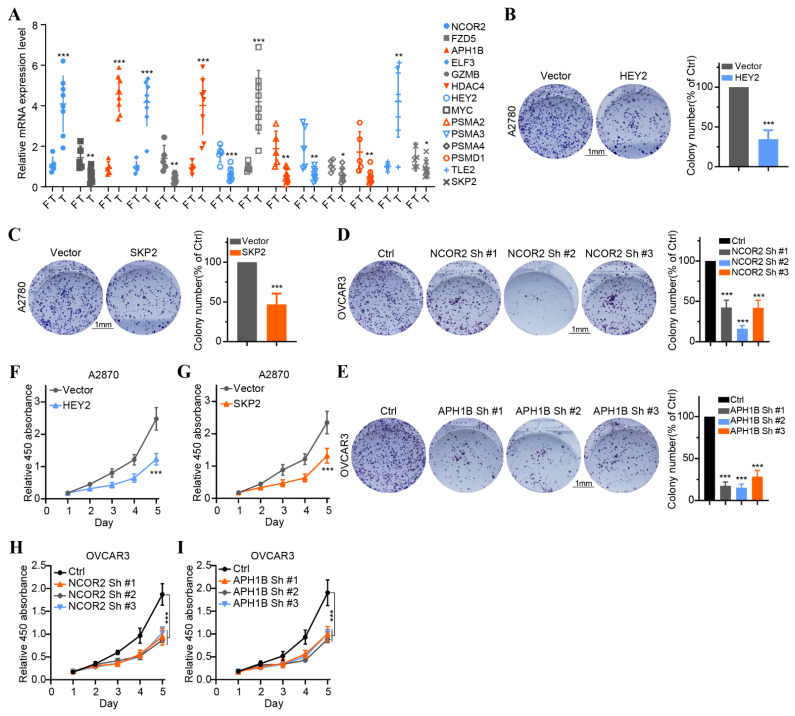
Validation of the expression of 14 overall survival-associated Notch genes in OC tissues and the effect of the four key genes on OC cell proliferation. (**A**) The expression of 14 overall survival-associated genes in OC and FT tissues was detected by RT-qPCR. (**B**,**C**) Colony formation assays of A2780 cells transfected with vector HEY2 and vector SKP2. (**D**,**E**) Colony formation assays of OVCAR3 cell transfected with sh-NC, NCOR2-sh1, NCOR2-sh2, NCOR2-sh3 and sh-NC, APH1B-sh1, APH1B-sh2, and APH1B-sh3. (**F**,**G**) CCK-8 assays of A2780 cells transfected with vector HEY2 and vector SKP2. (**H**,**I**) CCK-8 assays of OVCAR3 cell transfected with sh-NC, NCOR2-sh1, NCOR2-sh2, NCOR2-sh3 and sh-NC, APH1B-sh1, APH1B-sh2, and APH1B-sh3. * *p* < 0.05, ** *p* < 0.01, *** *p* < 0.001.

**Figure 10:**
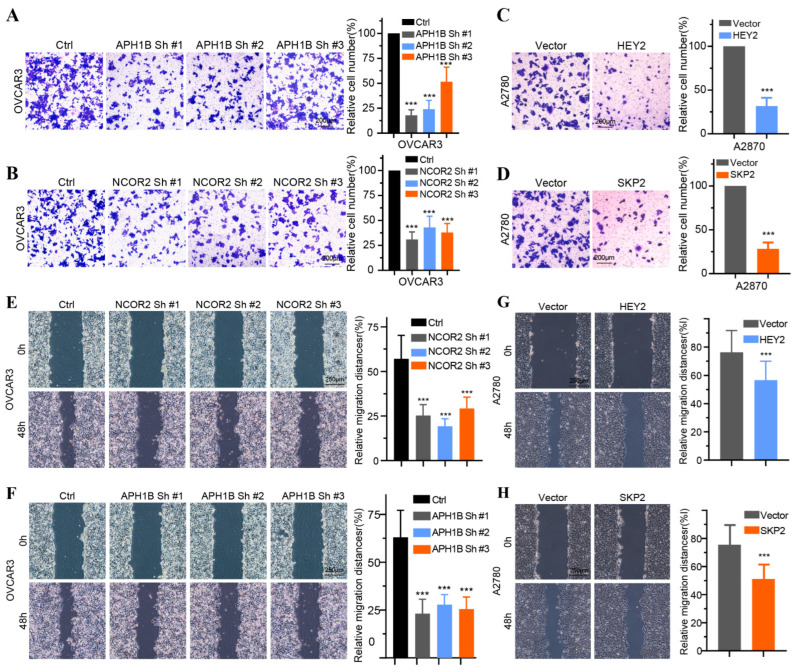
The effect of the four key Notch genes on OC cell proliferation, invasion, and migration. (**A**,**B**) Invasion assays of OVCAR3 cell transfected with sh-NC, NCOR2-sh1, NCOR2-sh2, NCOR2-sh3 and sh-NC, APH1B-sh1, APH1B-sh2, and APH1B-sh3. (**C**,**D**) Invasion assays of A2780 cell transfected with vector HEY2 and vector SKP2. (**E**,**F**) Wound healing assays of OVCAR3 cells transfected with sh-NC, NCOR2-sh1, NCOR2-sh3 and sh-NC, APH1B-sh1, APH1B-sh2, and APH1B-sh3. (**G**,**H**) Wound healing assays of A2780 cell transfected with vector, HEY2 and vector, SKP2. *** p < 0.001.

To validate the findings of database analysis, we measured the baseline mRNA expression levels of 14 key NTGs in OC tissues and FT tissues. Results indicated that NCOR2, APH1B, ELF3, HDAC4, MYC, and TLE2 exhibited higher expressions, FZD5, GZMB, HEY2, PSMA2, PSMA3, PSMA4, PSDM1, and SKP2 exhibited lower expressions in HCC patients (Figure 9A). Furthermore, we verified the effect of four vital NTGs (NCOR2, APH1B, HEY2, and SKP2) on cell proliferation, invasion, and migration in OC. After HEY2 and SKP2 overexpression, we found that the number of cells in HEY2 and SKP2-transfected A2780 cell groups was significantly lower compared to that found in the vector (Figure 9B,C,F,G). These results confirm that HEY2 and SKP2 markedly suppress proliferation in OC cells. CCK-8 assays and colony formation assays showed the knockdown of NCOR2 and APH1B significantly inhibited cell proliferation and colony-formation abilities in OVCAR3 (Figure 9D,E,H,I). We then evaluated the invasion and migration capacity of A2780 and OVCAR3-transfected cells via in vitro Transwell migration and wound healing assays. We further found that the knockdown of NCOR2 and APH1B inhibited the invasion and migration ability of OVCAR3 cells (Figure 10A,B,E,F). Furthermore, Transwell and wound healing assay results show that cell invasion and migration were significantly reduced in cells transfected with HEY2 and SKP2 compared with NC cells (Figure 10C,D,G,H).

## 4. Discussion

The Notch signaling pathway is an important signaling pathway in cells and is considered a major participant in the interaction between cancer cells and the TME [20,21,22,23]. In T-cell acute lymphoblastic leukemia, Notch activation mutations disarm cell cycle progression, inhibit apoptosis, and affect tumorigenesis [24]. Moreover, the abnormal activation or dysregulation of Notch signaling has been reported in CRC, breast cancer, prostate cancer, and other malignant tumors [25,26,27]. Notch can act as an oncogene or a tumor suppressor gene in different cancers or different cell populations of the same cancer [28]. However, few studies on the role of NTGs in OC have been conducted, and the correlation of NTGs with immunotherapy and OC prognosis remains unclear. This role was elucidated in this study.

In the current study, remarkable differences in NTG expression were found between OC tissues and normal ovarian tissues. PCA indicated that Notch might indicate the biological grouping of OC tumor tissue. Previous studies also confirmed that increased Notch gene copy number in OC tissues is associated with OC progression [29,30,31,32]. The dysregulation of Notch signaling can affect tumor proliferation, immune response, drug resistance, and prognosis [33,34]. However, their relationship in OC is rarely clarified. Therefore, we conducted an in-depth analysis of the correlation between NTGs and OC prognosis and found that most genes in the OC samples had some degree of amplification or deletion variation. *PSMA7*, *HDAC2*, and *PSMB5* mutations were related to OS, but further confirmation was needed to expand the sample size of mutations. Notch signaling dysregulation affects the development and prognosis of OC to a certain extent. In addition, 14 NTGs that were remarkably associated with OC prognosis were screened and analyzed by KM survival analysis. The result showed that the high expression of *GZMB*, *FZD5*, *PSMA4*, and *PSMA2* and the low expression of *APH1B* and *NCOR2* were remarkably associated with good OC prognosis. On this basis, Lasso regression analysis was combined to develop an NTRS model consisting of 11 NTGs to predict clinical outcomes in patients with OC. The prognostic model showed an excellent ability to classify patients into low- and high-risk groups. We found that patients in the low-risk group exhibited good outcomes, and evidence supports the stratification of risks in our model [35]. Notch is a target of tumor-mediated immunosuppression, and Notch reactivation in T cells may protect them from tumor-mediated immunosuppression and enhance their antitumor activity [36]. Therefore, NTRS can be used as an independent prognostic indicator for patients with OC.

Clinical data suggest that immunotherapy response is limited, and assessment based on sensitive tumor biomarkers may improve the predictability of immunotherapy response. This study reported that patients in the low NTRS group had a better prognosis in immunotherapy. The relationship between NTRS and tumor immunity, as well as the possible mechanism of predicting the prognosis of immunotherapy, was further elucidated. Correlation analyses of immune cell abundance, differential expression of immune checkpoint genes, immune characteristics, and immune score in the high and low NTRS groups were performed. CD8+ T cells, memory CD4+ T cells, T follicular helper cells, and M1 macrophages in the low-risk group were higher than those in the high-risk group, whereas monocytes and M2 macrophages in the high-risk group were significantly higher than those in the low-risk group. Immune cell recruitment plays a key role in cancer recognition and eradication. Favorable immunotherapy outcomes in multiple cancer types are associated with a higher proportion of tumor-infiltrating lymphocytes (TILs) [37,38]. TILs are considered a morphological manifestation of an anticancer immune response, especially CD4+ and CD8+ T cells and their immunomodulatory cytokines, which play a role in adaptive immunity [39]. CD8+ T cells are cytotoxic lymphocytes; their tumor–antigen interaction is through the production of IFN-γ, tumor necrosis factor α, granulocyte colony-stimulating factor, and other cytokines directly or indirectly lyse tumor cells. In comparison, CD4+ cells are usually not cytotoxic but can recruit and activate other cells, such as macrophages, B cells, dendritic cells, inflammatory cells, and other T cells [40,41,42]. Iwahashi found that the presence of CD8+ lymphocytes in ascites tumor cell populations is associated with good immune response status and prognosis in patients with high-grade serous carcinoma. T follicular helper cells play a protective role in nonlymphoid tumors and reduce immunosuppression [43]. M1 macrophages secrete and release proinflammatory cytokines, effector molecules, and chemokines to activate immune responses and play an antitumor role. M2 macrophages inhibit antitumor immune responses in various ways and are associated with poor disease outcomes [44,45].

This study showed the remarkable differences in gene expression at immune checkpoints between the two groups. PD-L1 (CD274), PD-L2 (PDCD1LG2), CTLA4, CD80, and CD86 were highly expressed in the low NTRS group. ICIs inhibit tumors that cause death by blocking receptor–ligand binding, upregulating immune function, and restoring T-cell cytotoxicity [46,47]. Tumor-derived PD-L1 or PD-L2 eventually inhibits the immunity of T cells, which can recognize and destroy tumors by binding to PD-1 [48]. CTLA4 acts as an inhibitory, costimulatory molecule that interferes with T-cell activation [49,50]. The natural ligands of CTLA-4 are CD80 (B7-1) and CD86 (B7-2), expressed by antigen-presenting cells (B cells, dendritic cells, and macrophages) and T cells [51]. CTLA4 reverses T-cell hypersensitivity, resulting in an antitumor response [52]. High CD80 expression in tumors leads to antitumor immunogenicity [51,53]. Singh et al. integrated molecules containing the extracellular domain of CD80 into the cell surface of tumor cells, which enhanced the immune response in gynecological cancer cell lines and lymphoma mouse models [54]. Consistent with previous studies, patients with high Notch signaling mutations have higher levels of immune checkpoint molecules, such as PD1 (PDCD1), PD-L1 (CD274), PD-L2 (PDCD1LG2), B7-H3 (CD276), and LAG3 [55]. In addition, NTRS and immune score had a remarkable negative correlation. Immunoscore influences the prognosis of many cancers [56,57,58,59]. Pages et al. highlighted the powerful prognostic impact of immune scores on patients with stage I–III colon cancer [60]. Two large cohort studies also showed that patients with stage III colon cancer and higher immune scores benefited more from chemotherapy than those with lower immune scores [61,62]. Therefore, activated immune cells, upregulated immune checkpoint molecules, and high immunogenicity may be the factors that contribute to a good prognosis in patients with OC with low NTRS who receive ICIs. These results suggest that the NTR model may be a potential predictor of OC prognosis and ICI acceptance, which is conducive to accurate and personalized treatment to prolong survival time.

Additionally, the expression level of the 14 NTGs in our signature was verified via RT-qPCR assay in OC tissues. The expression trend was consistent with the prediction of the above bioinformatics research. Notably, four vital NTGs (NCOR2, APH1B, HEY2, and SKP2) were closely related to the occurrence and development of OC. High NCOR2 in the tumors of patients with breast cancer predicted chemotherapy refractoriness, tumor recurrence, and poor prognosis [63]. Previous studies have shown that High-risk HPV E7 maintains stemness in cervical cancer stem-like cells via APH1B [64]. The expression of HEY2 protein was upregulated in HCC. HEY2 overexpression inhibited TGF-β-induced growth arrest [65]. At the same time, HEY2 is a promising candidate biomarker for molecular characterization and prognosis of CRC metastasis [66]. SKP2 is an essential component of the SKP2-SCF E3 ligase complex, which can bind k48 and K63-linked ubiquitin chains on different substrates to induce proteasomal mediated proteolysis or regulate the function of labeled substrates, respectively. SKP2 is overexpressed in various human cancers and is associated with poorer survival and poor therapeutic outcome, suggesting a role for SKP2 in tumorigenesis [67]. However, the exact molecular mechanisms of OC controlled by these four vital NTGs need more in-depth investigations. According to currently searchable literature, this study is the first to comprehensively establish an NTR prognostic model and draw prognostic correlation graphs for patients with OC. However, this study has limitations. First, although the risk profiles were established based on NTGs, the relationship between its members and prognosis has not been reported. Second, the correlation analysis between NTRs and immunotherapy needs to be validated in an independent cohort. Finally, single-cell sequencing is necessary to compensate for the limitations of traditional high-throughput sequencing and reduce the loss of much low-abundance information in the overall characterization.

## 5. Conclusions

In summary, the results of this study indicated that NRGs are strongly associated with OC. NTRS can be used as an effective biomarker for predicting the prognosis and immunotherapy response of OC. This study laid a foundation for further research on the relationship between NTGs and OC immunity.

## Data Availability

The datasets analyzed in the present study were from the public database. The data can be downloaded from: https://www.ncbi.nlm.nih.gov/geo/query/acc.cgi?acc=GSE26712 (accessed on 11 October 2021).

## Supplementary Materials

The following supporting information can be downloaded at: https://www.mdpi.com/article/10.3390/medicina59071277/s1, Figure S1: Nomogram establishment and performance verification. (A) Nomogram combined with age and NTRS. (B) 1−year, (C) 3−year, and (D) 5−year calibration diagrams. (E) ROC curve.

## Abbreviations

OC: ovarian cancer, OS: Overall Survival, NTRS: Notch risk score, NTGs: Notch genes, NTR: Notch risk, PFS: progression-free survival, ICIs: immune checkpoint inhibitors, TME: The tumor microenvironment, HNSCC: head and neck squamous cell carcinoma, CRC: colorectal cancer, NSCLC: non-small-cell lung cancer, CNVs: copy number variations, TCGA: The Cancer Genome Atlas, GEO: Gene Expression Omnibus, GTEx: Genotype-Tissue Expression, ICGC: International Cancer Genome Consortium, PCA: Principal component analysis, KM: Kaplan–Meie, ssGSEA: single-sample gene set enrichment analysis, FT: fallopian tube, FBS: fetal bovine serum, NCOR2: nuclear receptor corepressor 2, FZD5: Frizzled 5, GZMB: Granzyme B, HDAC4: histone deacetylase 4, PSMA2: Proteasome subunit alpha type 2, PSMA3: proteasome 20S subunit alpha 3, PSMA4: proteasome 20S subunit alpha 4, PSMD1: proteasome 26S subunit, non-ATPase 1, TLE2: the transducin-like enhancer of split 2, ROC: receiver operating characteristic, AUC: area under the ROC curve TILs: tumor-infiltrating lymphocytes.
