# Supplementary material for: A Novel Notch-Related Gene Signature for Prognosis and Immune Response Prediction in Ovarian Cancer
Source: Medicina (Kaunas). 2023 Jul 9;59(7):1277. doi: 10.3390/medicina59071277 (PMC10385113; doi:10.3390/medicina59071277)
Supplement: Supplementary file 1 [file medicina-59-01277-s001.zip › medicina-2411976-supplementary.pdf]

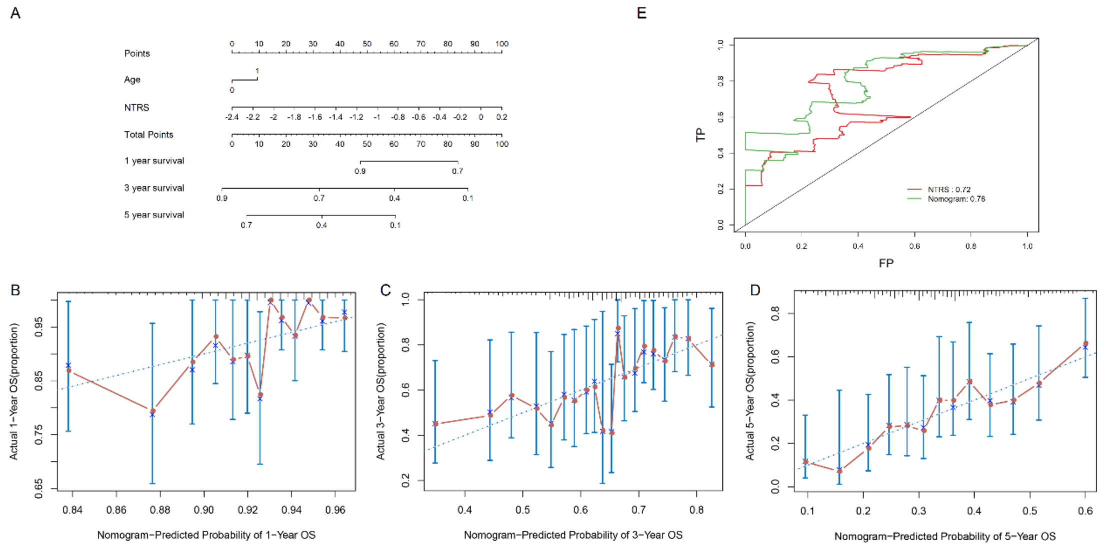

Supplementary Figure S1 Nomogram establishment and performance verification. (A) Nomogram combined with age and NTRS. (B) 1-year, (C) 3-year, and (D) 5-year calibration diagrams. (E) ROC curve.
